# Hemiballismus as the Initial Manifestation of Brain Metastasis: Diagnostic Discordance Between PET-CT and MRI in a Patient With Multiple Neoplasms

**DOI:** 10.7759/cureus.96684

**Published:** 2025-11-12

**Authors:** Leonardo D Paganella, Andrei L Schuster, Stefano S Kuiava, Natalia T Giudice da Costa, Gusthavo A Assmann Osaida, Antônio L Pacheco, Teo R Campos, Thiago L Dutra, Gustavo M Almeida

**Affiliations:** 1 Emergency Medicine, Hospital de Aeronáutica de Canoas, Canoas, BRA; 2 Primary Care, Hospital de Aeronáutica de Canoas, Canoas, BRA; 3 School of Medicine, Universidade do Vale do Rio dos Sinos, São Leopoldo, BRA; 4 Pediatric Neurology, Universidade Federal de Ciências da Saúde de Porto Alegre, Porto Alegre, BRA; 5 Neurology, Hospital de Aeronáutica de Canoas, Canoas, BRA; 6 Internal Medicine, Universidade Federal do Paraná, Curitiba, BRA

**Keywords:** brain metastases, hemiballismus, magnetic resonance imaging, movement disorders, multiple primary malignancies, neuro radiology, positron-emission tomography, subthalamic nucleus

## Abstract

Brain metastases are a frequent cause of intracranial tumors in adults and usually present with neurological deficits such as headache, seizures, or motor impairment. Hyperkinetic movement disorders, including hemiballismus, are exceptionally uncommon in this setting. Hemiballismus results from dysfunction within the subthalamic or neighboring basal ganglia regions and can occur secondary to various pathological processes. We report the case of an 80-year-old man with a history of ischemic stroke, myocardial infarction, multiple primary malignancies with hepatic metastases, and chronic kidney disease who presented with acute-onset left-sided hemiballismus predominantly involving the upper limb, accompanied by hematuria. A recent fluorodeoxyglucose positron emission tomography (FDG PET)-computed tomography (CT) revealed a new hypermetabolic focus in the right basal ganglia, along with progressive hepatic lesions. Magnetic resonance imaging (MRI) and angiography demonstrated diffuse cerebral atrophy, chronic microangiopathy, and old lacunar infarcts, but no new focal abnormalities. The discordance between PET-CT and MRI findings suggested a metabolically active lesion not yet detectable on structural imaging. During hospitalization, the patient developed transient delirium managed conservatively and was discharged clinically stable, without recurrence of involuntary movements. This case highlights the value of integrating functional and structural neuroimaging in the assessment of atypical neurological presentations in patients with multiple malignancies. PET-CT may reveal early metabolic alterations preceding MRI-detectable changes, emphasizing the importance of comprehensive clinicoradiologic correlation for timely diagnosis and individualized management.

## Introduction

Brain metastases are the most common intracranial tumors in adults, occurring up to 10 times more frequently than primary central nervous system (CNS) tumors and affecting approximately 10-30% of adults with systemic malignancies and 6-10% of children [1]. The most frequent route of spread is hematogenous dissemination, with a predilection for richly vascularized regions such as the cerebral hemispheres, cerebellum, and pons. Clinically, the most common symptoms include headache (40-50%), focal neurological deficits such as hemiparesis, and seizures. 

Movement disorders, including hemichorea and hemiballismus, represent rare manifestations (<1% of all movement disorder cases) and are typically associated with lesions of the basal ganglia, particularly the subthalamic nucleus [2].

Hemiballismus is a hyperkinetic movement disorder characterized by abrupt, forceful, and irregular flinging or ballistic movements of large amplitude involving the contralateral arm and leg. These involuntary movements result from dysfunction of motor pathways within the opposite side of the central nervous system, most commonly affecting the subthalamic region [3].

Contrast-enhanced magnetic resonance imaging (MRI) remains the diagnostic modality of choice for evaluating brain metastases, with sensitivity reaching up to 95% for lesions larger than 5 mm, and is considered the gold standard for diagnosis [4]. However, MRI has certain limitations, particularly in distinguishing tumor recurrence from radionecrosis or in patients with pre-existing structural abnormalities such as atrophy or microangiopathy [5].

The 18F-fluorodeoxyglucose (18F-FDG) positron emission tomography (PET)-computed tomography (CT), widely used in systemic oncologic assessment [6], has a more limited role in detecting brain metastases due to its relatively low sensitivity (21-44%), which results from the high physiological glucose uptake of normal brain tissue. Nevertheless, PET-CT can serve as a valuable complementary modality, particularly when MRI findings are inconclusive or technically limited [7].

This case illustrates the diagnostic challenges and clinical relevance of integrating functional and structural neuroimaging techniques in the evaluation of atypical neurological symptoms in patients with multiple primary malignancies.

## Case presentation

An 80-year-old man with a medical history of ischemic stroke, acute myocardial infarction (with placement of two stents in the left anterior descending artery), urothelial bladder carcinoma treated non-surgically in 2017, prostate adenocarcinoma treated with prostatectomy, colon cancer with hepatic metastases treated surgically in March 2025, and chronic kidney disease, presented to the emergency department of a tertiary hospital with hematuria and new-onset left-sided hyperkinetic movements predominantly affecting the upper limb, which had begun one day earlier. He denied headache, dizziness, weakness, or other neurological symptoms.

During initial management, the patient received biperiden, after which he developed a decreased level of consciousness without the need for invasive ventilatory support-accompanied by bilateral mydriasis and aphasia, but without recurrence of extrapyramidal manifestations. Given his medical history and significant cardiovascular comorbidities, an acute ischemic stroke was initially suspected.

**Figure 1:**
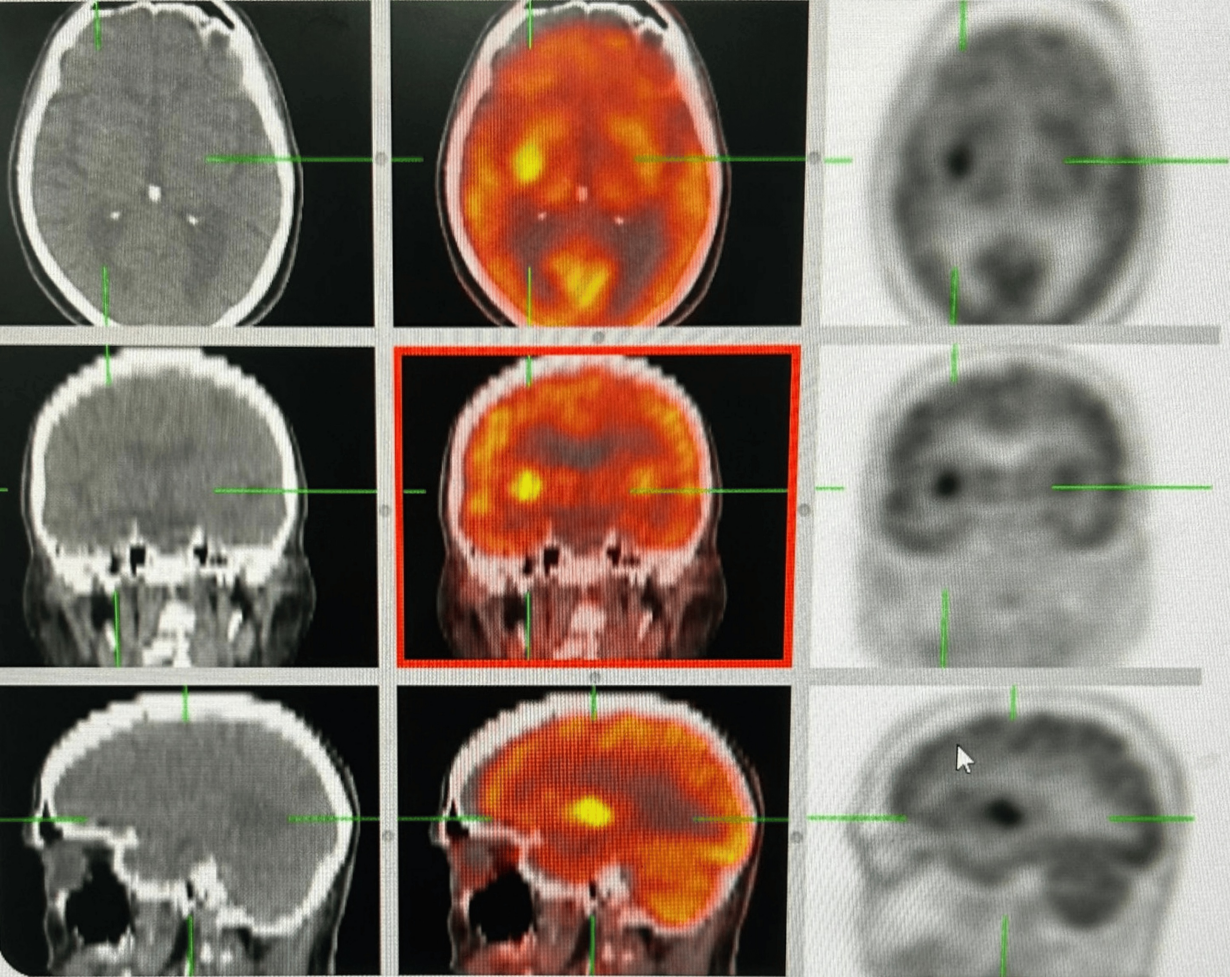
PET/CT demonstrating a new hypermetabolic focus in the right basal ganglia, compatible with recent disease activity.

A review of prior imaging revealed a PET-CT scan (Figure 1) performed two weeks earlier, which demonstrated a new hypermetabolic focus in the right basal ganglia, along with new hypermetabolic lesions in the hepatic parenchyma, findings suggestive of metastatic disease. These abnormalities were not present on a scan obtained six months earlier. Further evaluation with a brain MRI was subsequently recommended.

**Figure 2:**
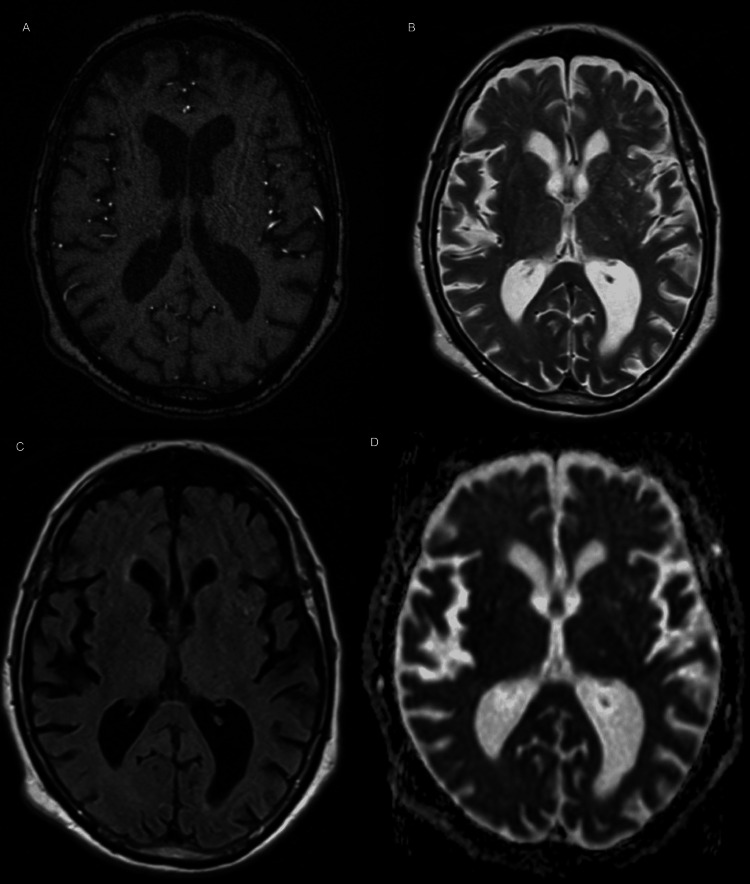
Axial magnetic resonance images: T1-weighted sequence (A), T2-weighted sequence (B), T2-FLAIR (C), and diffusion-weighted image (D), showing no evidence of lesions in the basal ganglia. FLAIR: fluid-attenuated inversion recovery

**Figure 3:**
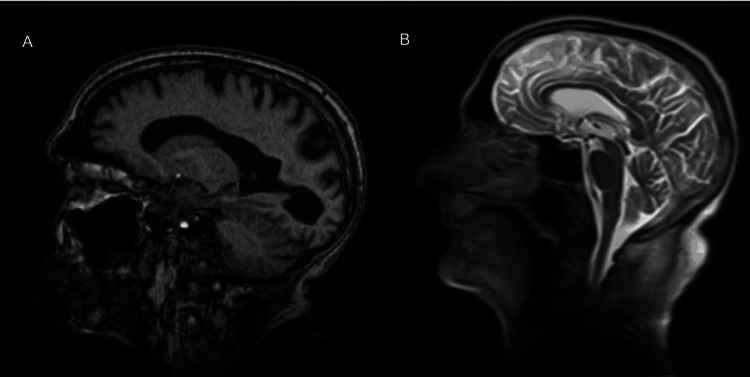
Sagittal magnetic resonance images: T1-weighted sequence (A) and T2-weighted sequence (B), showing no evidence of lesions in the basal ganglia.

**Figure 4:**
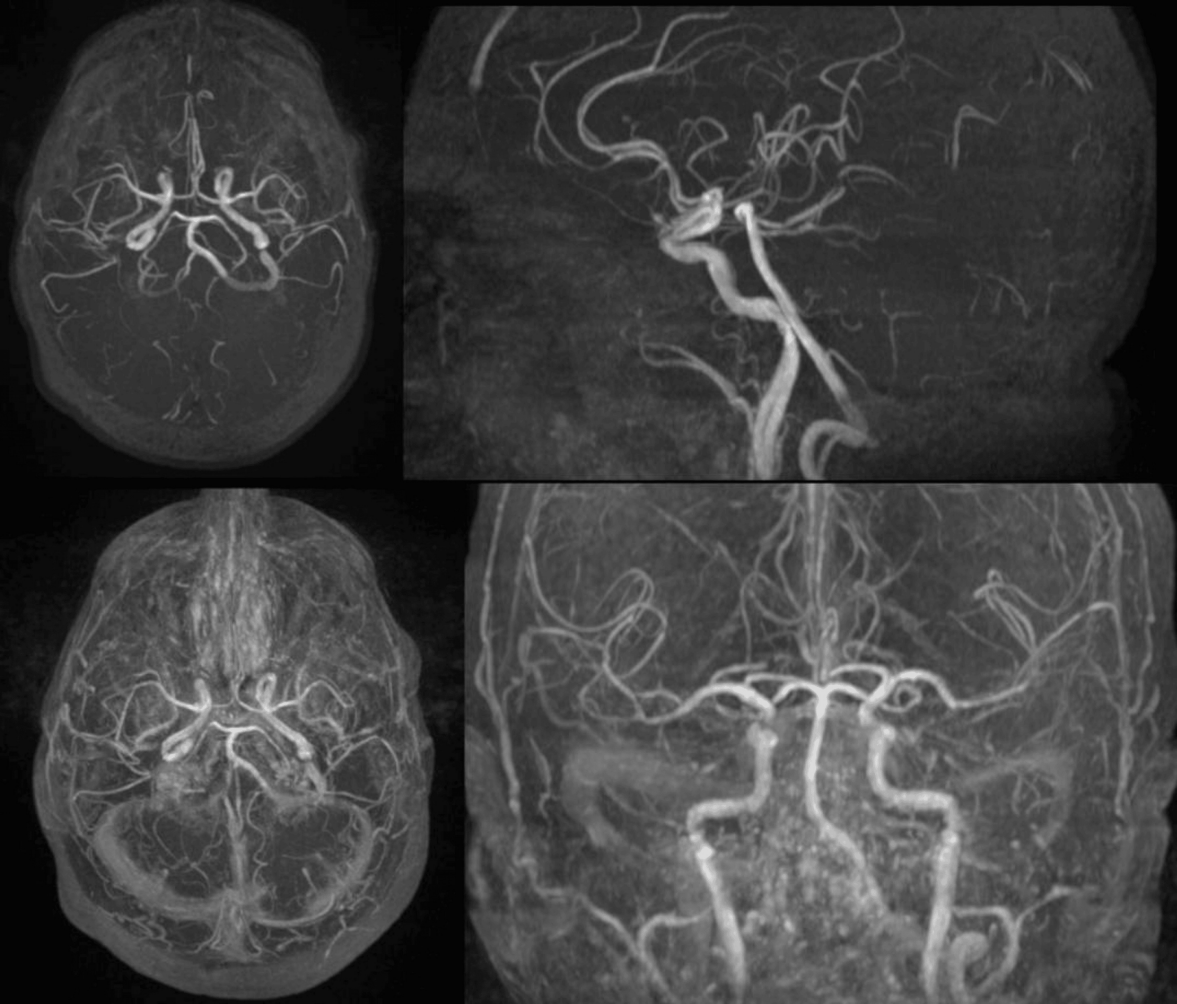
Brain magnetic resonance angiography shows no evidence of intracerebral masses or arterial thromboembolism.

Brain MRI (Figures 2, 3) and magnetic resonance angiography (MRA) (Figure 4) showed diffuse cerebral atrophy, including hippocampal volume loss, chronic microangiopathic changes, and old ischemic lacunar lesions. Arterial flow assessment was unremarkable. There was prominence of cortical sulci, Sylvian fissures, basal cisterns, and the ventricular system, consistent with diffuse cerebral atrophy. No new intracerebral masses or parenchymal lesions were identified.

Hepatic imaging revealed persistent lesions in the right hepatic lobe with peripheral enhancement and restricted diffusion: one in segment VI measuring 3.4 cm (previously 1.9 cm) and another spanning segments VI-VII measuring 4.7 cm (previously 2.7 cm). A new subcapsular lesion between segments VI and VII, 1.1 cm in diameter with peripheral enhancement and restricted diffusion, was also identified, consistent with a new secondary implant.

An acute ischemic stroke was ruled out, and the leading hypothesis became a new brain metastasis causing the focal neurological deficit. The patient was hospitalized in a general ward and developed hyperactive delirium during the first three days, but no further episodes of hemiballismus were observed. Delirium was managed conservatively with non-pharmacological measures and quetiapine 25 mg nightly.

Hematuria persisted without a history of local trauma. A Foley catheter was inserted, and the urology team was consulted. Cystoscopy revealed a hemorrhagic focus, which was cauterized; no local malignancy was identified. By the 10th day of hospitalization, the patient was alert, oriented, and exhibited no new focal neurological deficits. He was discharged for outpatient follow-up with neurology and oncology.

At the neurology clinic one week after discharge, he reported no recurrence of abnormal movements and had a normal neurological examination. However, he described a depressed mood and anhedonia. Vilazodone was initiated at 10 mg daily and increased to 20 mg after seven days. The neurology team scheduled a contrast-enhanced brain MRI with perfusion and magnetic resonance spectroscopy to further investigate possible brain metastasis and monitor lesion progression; results were pending at the time of this report.

The oncology team initiated pembrolizumab 400 mg every 42 days and continued to investigate the primary source of the suspected metastatic disease, which remained undetermined at the time of follow-up.

## Discussion

This case describes a patient with multiple primary malignancies who presented with isolated hemiballismus, an uncommon neurological manifestation associated with basal ganglia dysfunction [2]. Stroke and metabolic disturbances are the leading causes of hemiballismus, whereas neoplastic involvement is less frequent [3]. Given the patient’s acute focal symptoms, an ischemic event was initially suspected but subsequently excluded. Considering his oncologic background, a metastatic process became the most plausible explanation [1], supported by the imaging findings.

The hypermetabolic focus in the right basal ganglia identified on PET-CT corresponded anatomically to the contralateral motor symptoms [2]. The absence of structural abnormalities on MRI suggested either an early metabolically active lesion not yet apparent on morphological imaging or interpretative limitations related to chronic cerebral alterations such as atrophy and microangiopathy. Whereas MRI provides superior spatial and anatomical resolution, PET-CT captures underlying cellular glucose metabolism and can therefore detect functional disturbances that precede morphologic change. This fundamental distinction accounts for the apparent discordance between the two modalities observed in this case and reinforces the concept that functional imaging may reveal subtle metabolic abnormalities before the onset of structural alterations detectable by MRI [6,7].

Hemiballismus arises from the disruption of subthalamic circuits that modulate motor inhibition, leading to excessive excitatory output within the motor pathways. In this context, transient metabolic activity caused by neoplastic infiltration or local inflammation may explain the reversible nature of the symptoms observed in this patient [3].

Although MRI remains the mainstay for evaluating intracranial lesions [4], PET-CT provided complementary information that enhanced diagnostic confidence and guided further management [5]. The case highlights the value of integrating clinical evaluation with multimodal neuroimaging when approaching atypical neurological presentations in patients with complex oncologic histories.

## Conclusions

This case highlights the importance of a comprehensive clinical approach that integrates atypical neurological findings within the patient’s oncologic context. Although hemiballismus is an uncommon manifestation of brain metastasis, it should not be overlooked-particularly when functional imaging demonstrates new abnormalities, as shown by PET-CT in this case. It is also essential to consider and promptly exclude other potential causes of hemiballismus that pose a higher risk of acute deterioration and life-threatening complications, such as ischemic stroke, especially in patients with significant cardiovascular comorbidities.

The discordance between PET-CT and MRI findings illustrates the diagnostic challenges encountered in patients with chronic cerebral alterations. The complementary use of functional and structural imaging, combined with meticulous clinicoradiologic correlation, is crucial for the early detection of brain lesions and may facilitate more accurate and individualized management strategies. Furthermore, this case underscores that isolated neurological symptoms can precede structural abnormalities detectable on conventional imaging, emphasizing the need for heightened clinical vigilance in patients with multiple primary malignancies. An integrative and syndromic perspective remains essential for sound clinical reasoning and optimal patient care.
